# Clinical Implications of Exosomes: Targeted Drug Delivery for Cancer Treatment

**DOI:** 10.3390/ijms22105278

**Published:** 2021-05-17

**Authors:** Andrew E. Massey, Shabnam Malik, Mohammad Sikander, Kyle A. Doxtater, Manish K. Tripathi, Sheema Khan, Murali M. Yallapu, Meena Jaggi, Subhash C. Chauhan, Bilal B. Hafeez

**Affiliations:** 1 National Institute of Biomedical Imaging and Bioengineering (NIBIB), National Institutes of Health, Bethesda, MD 20892, USA; andrew.massey@nih.gov; 2 Department of Immunology and Microbiology, School of Medicine, University of Texas Rio Grande Valley, McAllen, TX 78504, USA; fnu.shabnam@utrgv.edu (S.M.); mohammed.sikander@utrgv.edu (M.S.); Kyle.Doxtater@utrgv.edu (K.A.D.); Manish.Tripathi@utrgv.edu (M.K.T.); sheema.khan@utrgv.edu (S.K.); mohan.yallapu@utrgv.edu (M.M.Y.); meena.jaggi@utrgv.edu (M.J.)

**Keywords:** exosome, tumor microenvironment, miRNAs, lncRNAs

## Abstract

Exosomes are nanoscale vesicles generated by cells for intercellular communication. Due to their composition, significant research has been conducted to transform these particles into specific delivery systems for various disease states. In this review, we discuss the common isolation and loading methods of exosomes, some of the major roles of exosomes in the tumor microenvironment, as well as discuss recent applications of exosomes as drug delivery vessels and the resulting clinical implications.

## 1. Introduction

Over the past few years, research into novel methods to deliver therapeutic agents for cancer has increased significantly. Various methods have been investigated, but one method that holds significant promise is the use of exosomal-based drug delivery. Exosomes are membrane-bound nanovesicles that are typically 30–150 nm in size with various bioactive molecules [1]. They are typically generated by first endocytosing various transmembrane proteins into endosomes within the cell, which are then sorted and form intraluminal vesicles. These vesicles are then released as the endosome merges with the cell membrane and releases its contents outside of the cell [2]. Tetraspanins (CD9, CD63, CD81) are one of the most common proteins expressed on the surface of exosomes and are often used as exosome-specific markers. These proteins have been shown to interact with different proteins such as integrins and major histocompatibility complexes (MHC). Exosomes commonly act as carriers of genetic and proteomic information, and are therefore vital in intercellular communication [3,4]. In its role as a cellular messenger, exosomes have been implicated in promoting cancer; because of this, they are also being investigated as potential therapeutic targets and delivery vehicles [5]. Because of their specific composition and ability to be loaded with various therapeutic payloads, it is plausible that exosomes can become a specific targeting system for drug delivery to cancer, even more so than targeted nanoparticle therapies currently seen in research.

## 2. Role of Exosomes in Cancer-Associated Microenvironment

**Figure 1:**
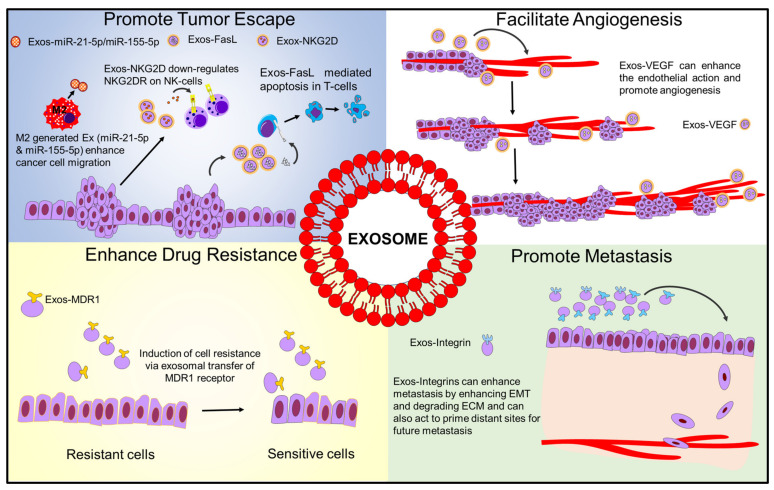
Schematic diagram showing how exosomes influence tumor microenvironment.

Regarding their pro-tumorigenic effects, exosomes are known to have multiple effects on the tumor microenvironment (TME). The TME consists of multiple components, including immune cells, fibroblasts, the extracellular matrix, basement membrane, endothelial cells, and cancer cells [6]. Considering all of these components, there are four major areas in which exosomes of various origins can affect the TME: promoting immune escape, drug resistance, enhancing metastasis, and promoting angiogenesis [7,8,9]. These effects are summarized in Figure 1.

### 2.1. Exosome-Mediated Immune Evasion

Regarding immune escape, tumor-associated exosomes have been shown to modulate multiple components of the immune system to prevent cytotoxic responses to cancer cells [10]. This can include effects such as impaired efficacy of NK cell receptor-mediated cytotoxicity, shifting macrophages to anti-inflammatory M2 phenotype, or inhibiting T cell function through the delivery of various lncRNAs or other molecules [11,12].

NK cells, otherwise known as natural killer cells, are lymphocytes associated with the innate immune system. They play a major role in defense against both infected cells and tumors. Unlike other immune cells, they are activated by a series of receptors, including NKG2D. Once activated, this receptor triggers most of an NK cell’s primary cytotoxic functions. One of the primary ways tumors can overcome this method of immune attack is through the release of exosomes with NKG2D ligands, which reduces the efficacy of this receptor-mediated cytotoxicity [13,14,15].

Tumor-associated exosomes have been shown to shift the phenotype of macrophages from M1 (pro-inflammatory, anticancer) to M2 (anti-inflammatory, tumorigenic) in multiple cancers [16,17,18,19]. In turn, exosomes derived from these transformed M2 macrophages can further enhance migration and invasion; this was demonstrated in recent work by Lan et al., where macrophage-derived exosomes were shown to contain high levels of miR-21-5p and miR-155-5p, leading to a downregulation of BRG1, a key factor in colorectal cancer metastasis [20].

These exosomes can also act to inhibit T cell function, and this can be accomplished in several ways. One method involves the binding of programmed cell death ligand 1 (PDL1) to PD1 receptors on T cell membranes, which leads to a reduced activation of its immune functions against cancer cells [21]. Other exosomal proteins, such as FasL, can even induce T cell apoptosis as shown by previous studies [22,23].

### 2.2. Exosomes Enhance Cancer Progression and Metastasis

Exosomes can enhance metastasis by enhancing EMT, promoting cell proliferation, or even degrading the ECM to promote cell invasion and metastasis from the primary site [24]. For example, recent work from Wang et al. demonstrated how cancer-associated fibroblast exosomes could transfer miR-181-5p to breast cancer cells, which inhibited CDX2 and led to an acceleration of EMT [25]. They can also act to prime distant sites for future metastases to grow—previous work by Hoshino et al. demonstrated that exosomes can localize at future metastatic sites, and the location of this could be determined in part due to the combination of integrins located on the exosomes [26]. Interestingly, recent work from Yuan et al. implied that breast cancer exosomes carrying miR-21 were more strongly associated with bone metastases than with non-metastatic cancers or other metastatic sites [27]. Degradation of the tumoral ECM was suggested in a recent study detailing breast cancer exosomes containing miR-4443 which inhibits tissue inhibitors of metalloproteinase 2 (TIMP2)—this study indicated that when these exosomes were secreted, there was an increase in metastasis as highlighted using a mouse model. When miR-4443 was suppressed, it led to reduced metastasis in vivo [28].

As outlined by multiple recent studies, exosomes can carry various biomolecules (including RNA, miRNA, proteins, or DNA) that can affect various signaling pathways in other cells. This can include autocrine/paracrine effects on cancer cells or transfer between cancer and stromal cells [29]. Recent research by Chen et al. demonstrated that exosomes secreted from bladder cells containing an lncRNA known as LNMAT2 led to lymphangiogenesis and increased metastasis through lymph nodes as indicated using a mouse model [30]. Furthermore, a recent study from Gao et al. highlighted that exosomes collected from drug-resistant breast cancer cells were rich in EphA2, a kinase that affects ERK signaling to promote cancer progression [31]. In a recent study by Vaidya and Sugaya, exosomal SOX2 DNA was analyzed as a potential biomarker of cancer progression in glioblastoma [32].

### 2.3. Exosomes and Drug Resistance

Exosomes can enhance drug resistance in cancer cells in various ways, whether by transmitting resistance markers to sensitive cells, or in a more direct manner by direct sequestration of chemotherapeutics [7].

Since the primary function of an exosome is to transfer information between cells, it is not surprising that this can include information on drug resistance to unexposed cancer cells. This has been extensively studied and has been shown to be a common function of cancer-associated exosomes. Although multiple methods have been discussed, one commonly seen technique employed via exosomal transfer is sending MDR1 to sensitive cells, which in turn can increase levels of efflux pumps such as p-glycoprotein (P-gp) [33,34,35].

Examples of the second method mentioned above have been shown in various cancers—for instance, previous work by Wang et al. demonstrated that breast cancer cells could promote resistance by directly sequestering adriamycin in exosomes, in addition to other resistance pathways. This was confirmed by UV spectrophotometry, where adriamycin was found localized within the exosomes generated by drug-resistant cells [36]. Another instance of this phenomenon was demonstrated by Federici et al. in melanoma; exosomes were capable of increased cisplatin uptake in an acidic environment, which also led to increased exosome production [37].

### 2.4. Exosomes Promoting Angiogenesis

Exosomes can also enhance the action of endothelial cells and promote angiogenesis—this has been demonstrated in multiple cancers [38,39,40,41,42]. Although multiple molecular targets have been identified, most of these in turn affect VEGF, which in turn leads to an enhanced angiogenesis at the tumor site. The pro-angiogenic effect of exosomes has also been shown in diseases outside of cancer, such as in heart disease [43].

### 2.5. Exosomal lncRNAs in Tumorigenesis

Long non-coding RNAs (a type of non-coding RNA >200 nucleotides in length) are known to lack protein coding potential but are still critical regulators of intercellular communication. LncRNAs can function as a decoy, guide, scaffold, adapter, or enhancer in various steps, including transcriptional regulation, resulting in various outcomes for cancer development [44]. LncRNAs from tumor cell-derived exosomes, called exosomal lncRNAs, can regulate the tumor microenvironment, inhibit immune cell function, promote growth and invasion of tumor cells, and impart drug resistance [45]. Moreover, lncRNAs can serve as potential diagnostic or prognostic biomarkers in different cancer types [46]. Carcinoma-associated fibroblasts (CAFs) were shown to promote stemness and chemoresistance in a previously published CRC mouse model by transferring exosomal H19 lncRNA, ref. [47] which in turn activated the beta-catenin pathway. LncRNA RUNX2-AS1, packed in multiple myeloma exosomes, has been shown to interact with transcription factor RUNX2, decreasing the osteogenic potential of mesenchymal stem cells [48]. LncRNA UCA1 present in exosomes from bladder cancer cells under hypoxic conditions was responsible for promoting EMT and reshaping the tumor microenvironment [49]. Exosomal lncRNAs were also shown to increase angiogenic factors and hence promote invasion and metastasis [50], as well as the pro-oncogenic CCAT2, POU3F3, and HOTAIR in glioma cells [51,52,53]. Bcl2 expression was increased with a decrease in Bax and caspase 3, indicating apoptosis inhibition. Pro-angiogenic genes VEGF-A, VEGF-D, IL-8, and angiogenin were found to be stimulated by lncRNA MALAT1 derived from epithelial ovarian cancer cell exosomes [54]. MALAT1 promotes cell proliferation in breast cancer and non-small cell lung cancer, ref. [55,56] while PCAT1 binds to miR-326 to promote cell proliferation in esophageal squamous cell carcinoma [57]. In colorectal cancer, 91H enhances metastasis by modifying HNRNPK expression [58]. In gastric cancer, ZFAS1 induces cell cycle, apoptosis, and EMT [59], while UCA1 has similar effects in bladder cancer [49]. LncRNA H19, FMR1-AS1, RUNX2-AS1, and Sox2ot have been studied for their roles in promoting tumor stem cells in different cancers [47,48,60,61]. H19 competes with miR-141 and activates the β-catenin pathway, maintaining tumor cell stemness and promoting drug resistance [47]. Exosomal lncRNA has also been shown to play a major part in drug resistance in different cancers. For instance, trastuzumab resistance has been reported in breast cancer by AGAP2-AS1 and SNHG14 [62,63], whereas UCA1 was responsible for tamoxifen resistance in breast cancer [64], cisplatin resistance in ovarian cancer [65], and cetuximab resistance in metastatic colorectal cancer [66]. In esophageal squamous cell carcinoma, Part1 produced gefitinib resistance [67]. Temozolomide resistance in glioblastoma was associated with increased lncRNA SBF2-AS1 function [68]. RP11-838N2.4 and H19 were found to be responsible for erlotinib and efitinib resistance in non-small cell lung cancer [69,70]. In renal cancer, ARSR enhanced sunitinib resistance [71]. Exosomal lncRNAs have also been studied for use as biomarkers in different cancers. MALAT1, PCAT-1, and SPRY4-IT1 were noted in one study to be highly concentrated in urine samples [72], whereas pCAT-1 and UbC H19 were upregulated in the serum [73,74] of bladder cancer patients. P21 was upregulated in the urine of prostate cancer patients [75]. An analysis of plasma has revealed many upregulated lncRNAs as biomarkers in different cancers, such as HOTAIR (breast cancer) [76], LNCV6 family (colorectal cancer) [77], SOX2-OT (lung squamous cell carcinoma) [78], as well as SAP30L-AS1 and SChLAP1 (prostate cancer) [79]. In cervical cancer, HOTAIR and MALAT1 were upregulated and MEG3 was downregulated within the cervicovaginal lavage in cervical cancer patients [80]. Serum analyses for biomarkers have revealed upregulation of MALAT1 in epithelial ovarian cancer [54], UEGC1 and HotTip (gastric cancer) [81,82], and HOTAIR (glioblastoma multiforme) [83]. LncRNA Gas5 was downregulated in serum collected from non-small cell lung cancer patients, suggesting its role as a tumor suppressor lncRNA [84]. Given the numerous actions that tumor-associated exosomes have on their microenvironment, cancer progression, and drug resistance, this leads to a strong case for repurposing these vesicles for therapeutic applications and utilizing this information for early biomarker analysis.

## 3. Common Isolation Methods of Exosomes

### 3.1. Ultracentrifugation

**Figure 2:**
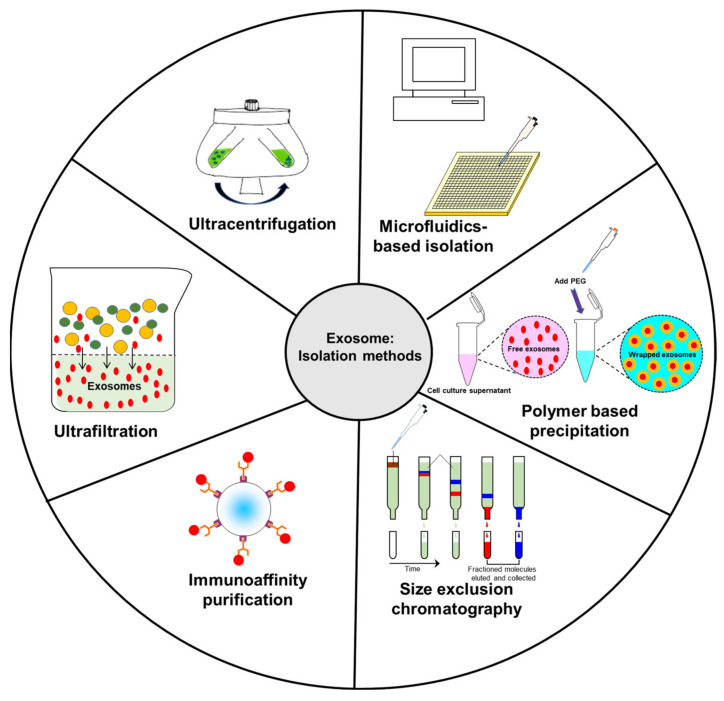
Schematic diagram representing common isolation methods for exosomes.

Ultracentrifugation is one of the most common methods used to isolate exosomes (Figure 2). Currently, this represents the “gold standard” for isolating exosomes through a series of centrifugation steps. Starting at lower speeds to remove cells and extraneous debris from the supernatant, the leftover fluid is then spun at higher speeds to make the exosomal pellet. Although this is a common method used to isolate exosomes, there is a concern that this method can lead to sample loss. Data suggest that repeated centrifugation can damage the vesicles and can lead to the possibility of co-sedimentation with highly immunogenic protein aggregates [85,86,87]. Lobb et al. also noted that repeated ultracentrifugation steps led to lower particle yields and reduced the overall recovery rate of exosomes [85]. While ultracentrifugation remains the current “gold standard” of exosome isolation, combination with other isolation methods can lead to an increase in the number of isolated exosomes.

### 3.2. Ultrafiltration

Ultrafiltration involves using a set of membranes to separate exosomes from proteins and other macromolecules. The exosomes are concentrated on the membrane, while proteins and other macromolecules are washed off. This procedure is thought to have higher exosome yields than ultracentrifugation [85]; however, it has some potential issues as well. It is possible that the exosomes or exosomal proteins can adhere to the membrane, which prevents them from being collected for further analysis or use [88]. Since additional force is applied to pass the analyzed liquid through the membranes, the exosomes risk being damaged in this procedure [85]. In a study by Alvarez et al., they raised the concern that certain proteins may not be properly filtered out, leading to possible difficulties with identifying key exosomal proteins for characterization [89].

One experiment conducted by Lobb et al. compared the effects of centrifugation and filtration on exosome yield and quality. It was shown that both processes yielded similarly sized particles ranging from 50 to 250 nm. Ultrafiltration, however, had a greater recovery of particles lower than 100 nm in size. It is also noteworthy that the procedure used for filtration was more time efficient. Concentrating 150 mL of cell-conditioned media by ultrafiltration took only 20 min, while it took two 90 min rounds of ultracentrifugation to concentrate the same volume of conditioned media [85].

Nordin et al. proposed a novel procedure to extract exosomes using ultrafiltration followed by liquid chromatography (UFLC). He then compared this to the “gold standard” of ultracentrifugation (UC). In brief, the conditioned media were collected after 48 h, and were then centrifuged for 5 min at 300× *g*, then 10 min at 1200× *g* to remove cellular debris. The resulting supernatant was then filtered using a 0.22 μm filter. The filtered supernatant was then purified using either UC or UFLC. The UFLC process led to higher yields of exosomes, and the biophysical properties of these exosomes were also better preserved than those purified via UC. The authors also noted that the procedure is scalable and adaptable to more complex biological media [90]. 

### 3.3. Immunoaffinity Capture

Immunoaffinity capture is a useful tool in exosome isolation, given that specific surface proteins can be identified to discriminate exosomes from other particles. It works primarily by the use of antibodies which selectively attach to specific proteins on the surface of exosomes to a type of filter, which can then be eluted out for further use [91]. One notable variant of this method used to rapidly isolate exosomes was an immunoisolation technique using magnetic beads. This process was demonstrated on LIM1863 colon cancer cell-derived exosomes using magnetic beads coated with anti-EpCAM. It was shown to have higher isolation efficiency than ultracentrifugation or density gradient separation [87].

### 3.4. Size Exclusion Chromatography (SEC)

**Figure 3:**
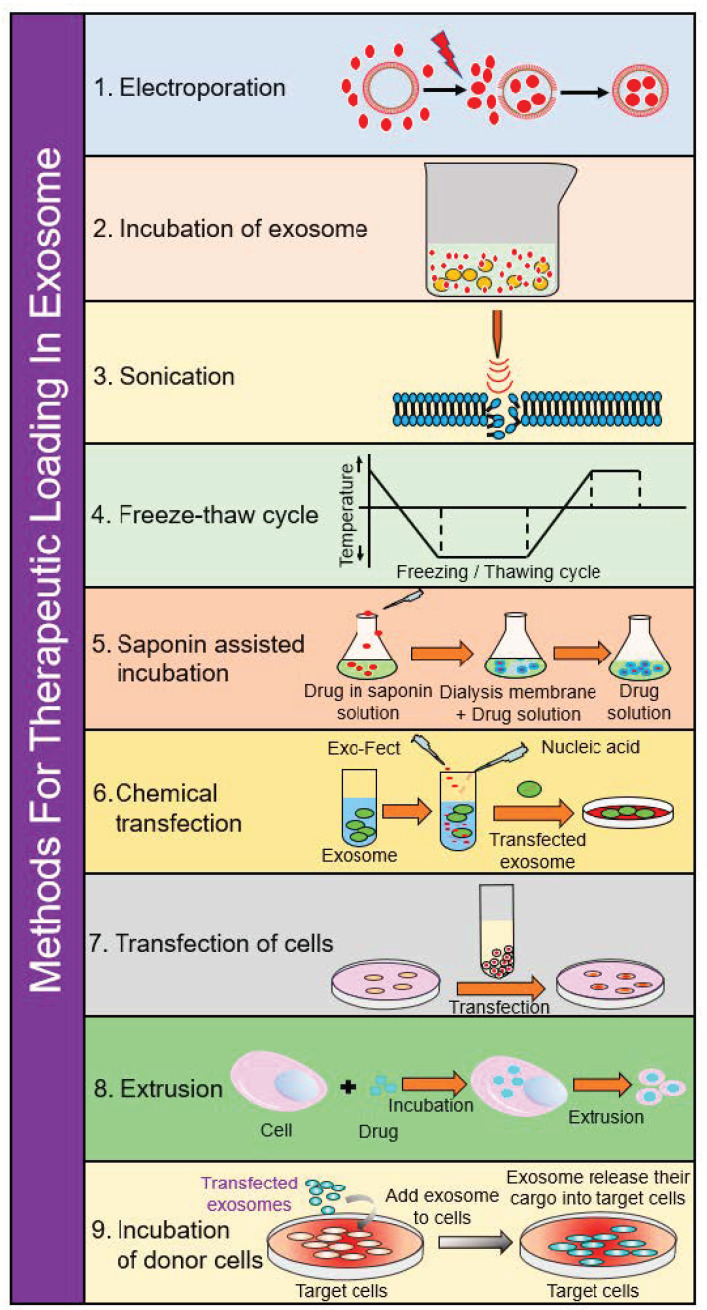
Schematic diagram depicting loading of various cargos in exosomes.

SEC takes a heterogeneous solution and separates components based on their size. To accomplish this, a column filled with porous beads is utilized as a filtration system—smaller components (such as exosomes or other small vesicles) can more readily pass through these pores, which increases their retention time within the column. By contrast, larger components are unable to pass through these pores and are more rapidly eluted from the column. Unlike ultracentrifugation, SEC can more readily preserve biological activity of extracellular vesicles as it is powered mainly by gravity flow, putting much less strain on the membranes of these vesicles. This method has also been shown to have highly sensitive and reproducible results in collecting exosomes. However, in part due to its gravity flow separation, it requires a long time to conduct this separation technique and is commonly used in conjunction with ultracentrifugation to further concentrate the final exosome sample [91,92,93]. A summary of this and the other loading steps can be found in Figure 3.

### 3.5. Polymer-Based Precipitation

Compared to the gold standard of ultracentrifugation, a precipitation method has been shown to potentially have higher purity and yield of exosomal RNA and protein. This, however, has only been confirmed from exosomes isolated from ascites [94].

Using urinary exosomes as a target, Alvarez et al. used a commercially available exosome precipitation reagent and a modified precipitation protocol to determine which isolation method showed the highest yield. Compared to the more common isolation methods, precipitation offered the highest yield of exosomes, microRNA, and mRNA. Although the authors noted that certain ultracentrifugation procedures were superior in regards to exosome isolation, overall the precipitation method was deemed a simple, fast, scalable alternative to isolate and identify exosomes [89].

### 3.6. Microfluidics-Based Isolation

It is possible to use “labs on a chip”, or microfluidics-based devices, to specifically catch exosomes via antibody-coated surfaces within the chip. The authors of one study noted that the use of a microfluidic approach was faster, cheaper, required less volume with fewer reagents, and could potentially isolate specific cell origin exosomes. They also noted that this method is compatible with clinical laboratory procedures as compared to current standards for exosome isolation. Moreover, it was noted with using microfluidics devices it is possible to sort exosomes from serum in one step as compared to magnetic bead-based systems [95].

## 4. Methods for Therapeutic Loading in Exosomes

### 4.1. Electroporation

Using an electric field, it is possible to create pores in the lipid bilayer of exosomes, which would allow for the entry of therapeutic agents. This procedure is favorable from a clinical standpoint since the procedures are easy to control. However, there are concerns that the membrane integrity of exosomes could be affected or that this procedure could lead to excessive aggregation. Electroporation also requires specific equipment to use, such as the Neon^®^ Transfection System as made by Thermo Fisher Scientific [96,97].

As discussed in an experiment conducted by Greco et al., HEK293 and MSC exosomes were suspended in electroporation buffer with various types of siRNA at a target concentration. The exosome-siRNA mixture was then transferred to a cuvette and electroporated using a Bio-Rad^®^ Gene Pulse XCell electroporation system. Using allophycocyanin-labeled siRNA, the authors were able to determine the loading efficiency of the exosomes. The exosomes loaded with siRNA were incubated with UMUC3 bladder cancer cells for 6 h. The authors found that there was a more than 28-fold increase in fluorescence intensity in the cell population with siRNA-loaded exosomes. The increased fluorescence intensity indicated that electroporation was successful in introducing siRNA into exosomes, and by extension into the bladder cancer cell [98]. In an experiment conducted by Zhang et al., the researchers used a modified calcium chloride transfection method and compared it to conventional electroporation, and it was noted that in both cases there was a similar level of miRNA introduction into exosomes [97].

### 4.2. Incubation of Exosomes

Perhaps the simplest method to incorporate therapeutic agents into exosomes is to simply incubate them in a solution with a high concentration of the target drug and allow for it to diffuse along the concentration gradient into the vesicles. This method may be more or less useful based on the hydrophobicity of the drug molecules in question [99]. Simple incubation of exosomes with the therapeutic cargo has been demonstrated with the use of curcumin-loaded exosomes. As seen in work by Zhuang et al., curcumin was mixed with exosomes in PBS, then incubated at 22 °C for 5 min. The samples were then subjected to a sucrose gradient centrifugation for 1.5 h at 36,000 rpm. Concentration of curcumin was determined using HPLC analysis. The authors of this study noted that using this loading technique led to promising results in vivo for brain inflammatory-related conditions [100].

### 4.3. Incubation of Donor Cells

In addition to incubating a solution of exosomes, a similar process could be accomplished using donor cells—a process where cells simply take up the drug of interest, then it generates drug-loaded exosomes which are collected. This method was demonstrated in research conducted by Pascucci et al., where mesenchymal stromal cells were treated with low-dose paclitaxel for 24 h then reseeded in fresh flasks. After growing, the media were collected and exosomes were isolated, which were found to incorporate paclitaxel and showed therapeutic effect on pancreatic cancer cells in vitro [101].

### 4.4. Saponin-Assisted Incubation

Saponin, a surfactant molecule, can be used to generate pores in the membrane of exosomes to increase their permeability to the therapeutic cargo being loaded. Since standard incubation is more effective with hydrophobic agents, this method may be useful to incorporate more hydrophilic drugs into an exosome. However, there is concern that saponin has a hemolytic effect, so its concentration should be kept to a minimum, and ideally should be purified from the final product [99,102,103].

### 4.5. Sonication

Using a probe sonicator, exosomes can be mixed with a drug to enhance uptake of the therapeutic cargo. Sonication leads to considerable deformation within the membrane, allowing for increased diffusion of drugs into the exosome [104]. This method has been shown to be effective in multiple studies—in some cases, it has led to multiple layers of drug encapsulation, with some incorporating inside of the exosome and some within the membrane, leading to a two-stage drug release, where the membrane-bound portion is released more rapidly, and the internalized drug is released over a longer timeframe. This was demonstrated in paclitaxel-loaded exosomes as highlighted in research by Kim et al., which also indicated a high drug loading efficiency and that sonication had minimal impact on the stability of exosomal lipid structure [105]. While this method may be useful for certain drugs, it was shown to be somewhat more problematic for nucleic acids, as the method was shown to lead to aggregation and/or degradation [106].

### 4.6. Extrusion

To use the extrusion method, exosomes are mixed with a drug, and the resultant mixture is passed through membranes with 100–400 nm size at a controlled temperature. This causes vigorous mixing of the exosomes and the drug, leading to membrane disruption and drug loading. Currently, the effects of the harsh mechanical forces induced by extrusion on exosomes are not fully understood [99,104]. However, at least one study has indicated that extrusion of exosomes leads to an altered zeta potential and causes cytotoxicity, while other loading methods did not cause cytotoxic effects [102].

### 4.7. Freeze–Thaw Cycling

In this method, drugs are mixed with exosomes at room temperature then rapidly frozen using liquid nitrogen or placement at −80 °C, followed by a rapid thaw back to room temperature. This process, as discussed in work by Sato et al., is repeated three times to ensure proper encapsulation. This work also highlighted the use of freeze–thaw methods to create exosome-mimetic liposomal particles [107]. In comparison to other incorporation methods, however, freeze–thaw cycling can cause exosomal aggregation and is typically less effective than sonication or extrusion in terms of drug encapsulation [99,104].

### 4.8. Chemical Transfection

Commercially available transfection reagents have been used to load exosomes with siRNA as shown in several studies [108,109]. This method may not be ideal due to a lower efficiency of loading as compared to electroporation. Moreover, use of the Lipofectamine 2000 reagent was noted to create micelles which could have altered the purity of the exosomal preparation. Due to these concerns, it is thought that chemical transfection of exosomes is not an efficient method of loading the drug in exosomes [96].

### 4.9. Transfection of Cells

Possibly the most common method of loading exosomes for targeted nucleic acid delivery is to transfect the donor cells with the gene target of interest, which can then be packaged into exosomes [96]. One example of this method was highlighted in a recent study by Katakowski et al., where bone marrow stromal cells were transfected with miR-146b and the exosomes were then collected to treat 9 L gilosarcoma cells. The resulting collected exosomes from the transfected cells showed increased miR-146b expression and were shown to reduce the growth of glioma in rat models [110].

## 5. Use of Exosomes in Targeted Delivery

**Figure 4:**
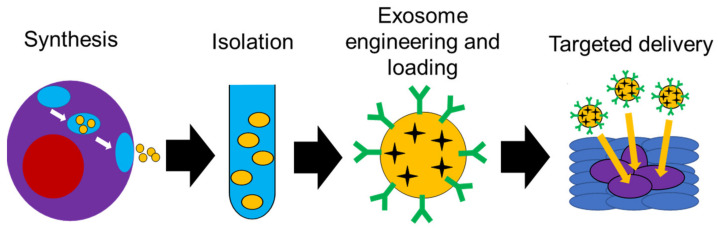
Schematic diagram showing possible use of exosomes in targeted delivery.

The natural designation of exosomes as cargo transporters between cells lends credence to the notion that exosomes are a naturally occurring, targeted delivery system. The use of exosomes as drug carriers hijacks this natural targeted delivery system to help improve therapeutic outcomes (Figure 4). An example of this was shown using exosomes isolated from HEK293 human embryonic kidney cells to deliver siRNA to bladder cancer cells. The exosomes were loaded with polo-like kinase 1 (PLK1) siRNA via electroporation and then co-cultured with UMUC3 metastatic bladder cancer cells. In an in vitro study, it was noted that the bladder cancer cells internalized the HEK293 exosomes more than normal bladder cells, which led to a successful knockdown of PLK-1 mRNA and protein [98].

As discussed by Ha et al., common NP delivery systems include liposomes and polymeric NPs. Liposomes can ideally evade the host’s immune system and have a long circulating capability as well as high stability. Polymeric NPs currently are more stable than liposomes, but there are still concerns with biocompatibility and long-term safety. Exosomes are therefore highly desirable due to long circulating half-life, intrinsic ability to target tissue, biocompatibility, and at most minimal toxicity. In this regard, they have been widely researched [111].

In one example of drug delivery, exosomes were loaded with curcumin to treat inflammatory disease. The exosomes formed a complex with curcumin which enhanced its efficacy when compared to free curcumin in a clinical trial. In one study, curcumin was loaded on EL-4 murine tumor cell-derived exosomes via the incubation method. Markers were then used to identify the curcumin–exosome complex (including CD81). Experiments in vitro showed that exosomal curcumin significantly reduced inflammatory cytokine levels compared to native curcumin. An in vivo mice model also showed enhanced survivability in lipopolysaccharide-induced septic shock for mice treated with exosomal compounds [112,113,114].

Exosomes were also noted to enhance blood brain barrier (BBB) penetration. Although other methods have been investigated to improve the CNS delivery of various drugs, such as the use of nano-formulations and the addition of PEG to the structure, there are still potential issues to overcome with these methods (e.g., rapid clearance by mononuclear phagocyte system and reduced distribution in the brain, respectively) [115,116].

The anticancer applications of exosomes have also been investigated. One such study shows paclitaxel and doxorubicin being encapsulated into exosomes. After the exosomes were isolated, loaded with chemo drugs via incubation, and characterized, they were then tested in vivo using a zebrafish model. The loaded exosome systems showed significantly improved CNS delivery capacity compared to base drug [117]. Further of note, since the exosomes were small and native to the animal, they inherently avoided phagocytosis, which led to a reduced immune response [111].

Recently, Qambrani et al. highlighted that cancer-cell-derived exosomes could be used as both a drug delivery system and as a potential fluorescent biomarker, as shown with HeLa-derived exosomes loaded with doxorubicin and silver nanoclusters [118]. In a recent study from Zhou et al., exosomes derived from bone marrow mesenchymal stem cells were loaded with siRNA and oxaliplatin and were used as an experimental treatment for pancreatic cancer in a mouse model. Their results indicated an increased uptake of these agents with exosomal delivery, indicating a greater therapeutic effect as compared to free drug both in vitro and in vivo [119].

Recently, there has been significant interest in the application of bovine-milk-derived exosomes as a drug delivery vehicle. Several recent studies have been published showing the potential of using commercially available milk as a source of exosomes which can then be engineered for use as biocompatible nanocarriers for various therapeutic agents [120,121]. For instance, Li et al. recently showed that milk exosomes were first isolated, coated with hyaluronan for CD44-targeting, and loaded with doxorubicin, a process that significantly increased the uptake and therapeutic effect of this drug against cancer cells in vitro [122]. However, one recent study conducted by Carobolante et al. compared the uptake efficacy of milk-derived exosomes to Caco-2 epithelial cell exosomes, and they concluded that milk-derived exosomes had a less efficient uptake when compared to epithelial exosomes. Their results suggest that additional modifications would be needed for milk-derived exosomes to be used as an oral drug delivery vehicle [123]. Milk-derived exosomes have also been successfully used to deliver paclitaxel as shown in a previous in vivo study by Agrawal et al., where paclitaxel-loaded exosomes led to a greater inhibition of tumor growth than free paclitaxel, with a reduction in systemic side effects [124].

Exosomes have also been used to deliver therapeutic proteins in addition to small molecule drugs. A recent study showed exosomes loaded with the antioxidant catalase were able to cross the BBB and improve the clinical course of Parkinson’s disease. Catalase was added to the exosomes in several different ways (incubation, freeze–thaw cycles, sonication, and extrusion). Using a Western blot analysis, it was determined that sonication and extrusion had the best overall incorporation of catalase. In vivo mouse models showed significant distribution of the exosomes in the brain, which indicated an ability of the exosomes to properly deliver and target brain tissue [125].

As stated earlier, exosomes naturally carry nucleic acids such as DNA and RNA [3,4], and because of this they have been intensely investigated as a method of delivering genetic therapies, such as small interfering RNA (siRNA) and microRNA (miRNA) [111].

As a therapeutic agent, siRNA can be used to downregulate or disrupt the expression levels of target genes. Normally, siRNA has low stability and quickly degrades in systemic circulation. Exosomes act as a delivery vehicle to protect these RNA molecules from degradation in systemic circulation. They are ideal for this task, as they have the natural ability to deliver genetic material from cell to cell and are non-immunogenic to the patient [111]. One study conducted by Wahlgren et al. discussed the ability of delivering siRNA using exosomes. After isolating exosomes from lung cancer cells, exogenous siRNA was introduced into the exosomes delivered to human blood cells, demonstrating the potential of using exosomes for gene therapy [108]. Kamerkar et al. demonstrated the use of kRAS siRNA-loaded exosomes in pancreatic cancer mouse models—interestingly, when compared to siRNA loaded liposomes, the exosome group exhibited a greater reduction in tumor growth, as well as a reduced clearance from the body [126].

miRNA, a short form of non-coding RNA, binds to complementary sequences of mRNA to control post-transcriptional gene expression [127]. Since exosomes are known to naturally carry miRNA [111], they represent a prime delivery system for this type of gene therapy. A recent study by Mathiyalagan and Sahoo demonstrated the potential of exosomes to deliver miRNA into recipient cells in the heart for cardiovascular disease. They showed that CD34+ stem cell-sourced exosomes could be used to deliver pre-miR (mi-RNA) to regulate gene expression [128].

There is considerable interest in researching the drug delivery potential of exosomes in various diseases, especially cancer. As indicated by a recent search on PubMed, there are over 630 published articles using the search terms “exosomes AND cancer AND drug delivery”. Many of these articles have been published within the past three years. Table 1 highlights several recent studies demonstrating the various ways in which researchers seek to exploit exosomes as a natural drug delivery system.

## 6. Clinical Use of Exosomes: Progress and Promise

As a delivery system, exosomes hold significant promise with regards to specific targeting—it is feasible that a patient’s own cells could be collected to fabricate a delivery system that could be specific to that disease. This is primarily due to exosomes being a “natural delivery system,” as they are commonly used for intercellular communication. Moreover, due to their small size and origin, they can avoid phagocytosis and degradation by macrophages, allowing them to circulate for an extended time. Being derived directly from a patient’s cells, there is little (if any) risk of an immune response. They can also potentially avoid the endosomal pathway and lysosomal degradation unlike liposomes or polymeric nanoparticles. This allows exosomes to deliver agents directly to the cytoplasm, representing an enhanced delivery technique over currently available methods. Exosomes are naturally stable and, depending on their composition and source, have inherent targeting properties. These targeting properties can be tailored to specific cancers or other diseases with specific markers or proteins. They are also able to cross the BBB, which allows for delivery of CNS-active agents [111]. There is also extensive clinical effort to determine the use of exosomes in cancer—as seen on Clinicaltrials.gov, accessed on 4 May 2021, there are currently dozens of trails when searching for the terms “cancer” and “exosomes”—although most deal with the use of exosomes as a diagnostic marker, there are some with an express interest in the use of exosomes as a delivery system (such as trials NCT01294072 and NCT03608631).

Interestingly, there has also been research that intends to create “exosome mimetics” from liposomes. From a composition standpoint, the two structures are not dissimilar; both are composed of a lipid bilayer and can have specific proteins or markers embedded within its surface. It has been postulated that if certain proteins (e.g., tetraspanins, adhesion and targeting molecules, etc.) could be integrated into a liposome’s membranes, that they could in essence have similar properties to those of real exosomes. These mimics could potentially be used to overcome some of the current challenges that exist with exosome production [4].

Currently, there are still challenges with exosomes as a delivery system. Namely, the overall long-term safety and therapeutic effect are not well understood, as more thorough understanding and research are still required. Moreover, there are issues with purification and isolation techniques. One of the main methods of isolation, ultracentrifugation, is known to have low yields and can even damage exosomes due to the centrifugal forces applied to the vesicles. Although some of the other methods described above have been found to yield higher quality results, there is still the challenge of large-scale manufacturability that has not yet been solved [111].

## Figures and Tables

**Table 1:** Selected recently published articles highlighting strategies exploiting the use of exosomes as a drug delivery vehicle in cancer.

Source of Exosomes	Cargo	Cancer Type	Key Findings	Reference Number
293T cells	5-FU, miR-21i	Colorectal	Cell cycle arrest, reduced proliferation, increased apoptosis; reduced cancer growth in vivo with minimal toxicity	[129]
Bone marrow mesenchymal stem cells	Galectin-9 siRNA and oxaliplatin	PDAC	Enhanced drug uptake, reduction of M2-like macrophages and enhanced anti-tumor immunity, greater tumor reduction in vivo	[119]
Autologous pancreatic cancer cells (Panc-1) and heterologous lung cancer cells (A549)	Gemcitabine	Pancreatic	Enhanced uptake of autologous exosomes; reduced tumor growth compared to free gemcitabine; enhanced survival in vivo	[130]
MDA-MB-231 and HT-29 cells	miRNA 126	Non-small lung cell cancer	Enhanced uptake of MDA-MB-231 exosomes; reduction of proliferation and migration in vitro; reduced metastatic nodules in vivo with minimal toxicity	[131]
HeLa and L02 cells; exosomes isolated from blood cancer patient	Doxorubicin, silver nanoclusters and DNA	Cervical and blood cancers	Enhanced uptake of drug-loaded exosomes; superior theranostic capability compared to free silver nanoclusters	[118]
Cow milk and Caco-2 cells	Curcumin	Colorectal	Superior uptake noted with Caco-2-derived exosomes compared to milk-derived exosomes; enhanced therapeutic effect of CUR compared to free drug	[123]
Cow milk	Hyaluronan and doxorubicin	Breast, lung, kidney	Enhanced uptake into CD44 overexpressing cells, with superior cytotoxicity to free drug	[122]
Cow milk	Paclitaxel	Lung	Sustained release seen up to 48 h in vitro; significant growth inhibition after oral administration in vivo	[124]
Normal fibroblast-like mesenchymal cells	siRNA specific to oncogenic KRAS	Pancreatic	Superior KRAS targeting compared to liposomes; enhanced tumor suppression and survival in vivo	[126]
Umbilical cord macrophages	Cisplatin	Ovarian	Enhanced therapeutic effect of exosomal cisplatin in vitro against resistant and sensitive cell lines	[132]
Dental pulp mesenchymal stem cells	miR-34a	Breast	Downregulation of cancerous phenotype in vitro	[133]
Adipose tissue-derived mesenchymal stem cells	miR-199a	Hepatocellular carcinoma	Sensitized cancer cells to doxorubicin therapy both in vitro and in vivo	[134]
Mesenchymal stem cells	Doxorubicin	Colorectal	Significantly enhanced suppression of tumor growth in vivo compared to free doxorubicin	[135]
HEK293 cells	miR-34a	Pancreatic	Induced apoptosis in cancer cells in vitro; significant suppression of tumor growth in vivo	[136]
Bone marrow mesenchymal stem cells	miR-193a	Non-small cell lung cancer	Suppressed colony formation, proliferation, and invasion in vitro; reduced tumor volume in vivo	[137]
